# Letter to the editor regarding Extracorporeal membrane oxygenation for COVID-19: a systematic review and meta-analysis

**DOI:** 10.1186/s13054-021-03702-6

**Published:** 2021-08-09

**Authors:** Dominik Johannes Hoechter, Andrea Sabine Becker-Pennrich, Benjamin Peter Geisler, Bernhard Zwissler, Michael Irlbeck, Kollengode Ramanathan, Kiran Shekar, Ryan Ruiyang Ling, Ryan Barbaro, Graeme MacLaren, Eddy Fan, Daniel Brodie

**Affiliations:** 1 Department of Anesthesiology, LMU Klinikum, Ludwig-Maximilians-University Munich, Marchioninistr. 15, 81377 Munich, Germany; 2 Institute for Medical Information Processing, Biometry, and Epidemiology, Ludwig-Maximilians-University Munich, Marchioninistr. 15, 81377 Munich, Germany; 3 Massachusetts General Hospital, Harvard Medical School, 55 Fruit St., Boston, MA USA; 4 Comprehensive Pulmonary Center Munich (CPC-M), Member of the German Center for Lung Research (DZL), LMU Klinikum, University Hospital, Ludwig-Maximilians-University Munich, Max-Lebsche-Platz 31, 81377 Munich, Germany; 5 Yong Loo Lin School of Medicine, National University of Singapore, Singapore, Singapore; 6 Cardiothoracic Intensive Care Unit, National University Heart Centre, National University Hospital, Level 9, 1E Kent Ridge Road, Singapore, 119228 Singapore; 7 Adult Intensive Care Services and Critical Care Research Group, The Prince Charles Hospital, Brisbane, QLD Australia; 8 Queensland University of Technology, Brisbane, Australia; 9 University of Queensland, Brisbane, Australia; 10 Bond University, Gold Coast, QLD Australia; 11 Division of Paediatrics Critical Care Medicine, University of Michigan, Ann Arbor, MI USA; 12 Child Health Evaluation and Research Center, University of Michigan, Ann Arbor, MI USA; 13 Interdepartmental Division of Critical Care Medicine, University of Toronto, Toronto, Canada; 14 Department of Medicine, Columbia University College of Physicians and Surgeons, New York, NY USA; 15 Center for Acute Respiratory Failure, New York-Presbyterian Hospital, New York, NY USA

**To the Editor,**

We read with great interest “Extracorporeal membrane oxygenation for COVID-19: a systematic review and meta-analysis” by Ramanathan et al. and appreciate their diligent work and their conclusion to offer extracorporeal membrane oxygenation (ECMO) therapy to carefully selected patients presenting with severe acute respiratory distress syndrome (ARDS) related to COVID-19 [1]. However, the relatively low calculated mortality of 37.1% caught our attention.

While reviewing the input data in detail, we noticed two discrepancies: First, the data from the Japanese National Database by Takeda in Table 1 of their paper is stated to contribute 237 patients, while Figure 2 mentions 370 patients and the supplemental figures mention still different numbers of patients. Second, the numbers of survivors in Figure 2 seem to suggest that some patients had not yet been discharged and some were still on ECMO. As eleven of the 22 studies reported on patients still receiving care in hospital or even being on ECMO with a percentage ranging up to as high as 58% of the total number of patients, we see the risk of underreporting the true mortality and conveying a possibly too optimistic picture.

We recalculated mortality without taking patients into consideration who are or were still being treated as well as excluding studies reporting on patients not yet discharged using the R software version 4.0.3 with the “meta” package version 4.18-2 and the same parameters as used by Ramanathan et al. The resulting forest plots are depicted in Fig. 1. We calculated the pooled mortality as 41.4% with a 95% confidence interval of 34.8% to 48.2% and 41.1% (95% CI 32.3–50.2%), respectively. For context, in-hospital mortalities of larger COVID-19 cohorts treated with ECMO have been reported between 45.9% and 53.0%, with advanced age being associated with higher mortality which Ramanathan et al. also found in their meta-regression [2, 3].

**Fig. 1:**
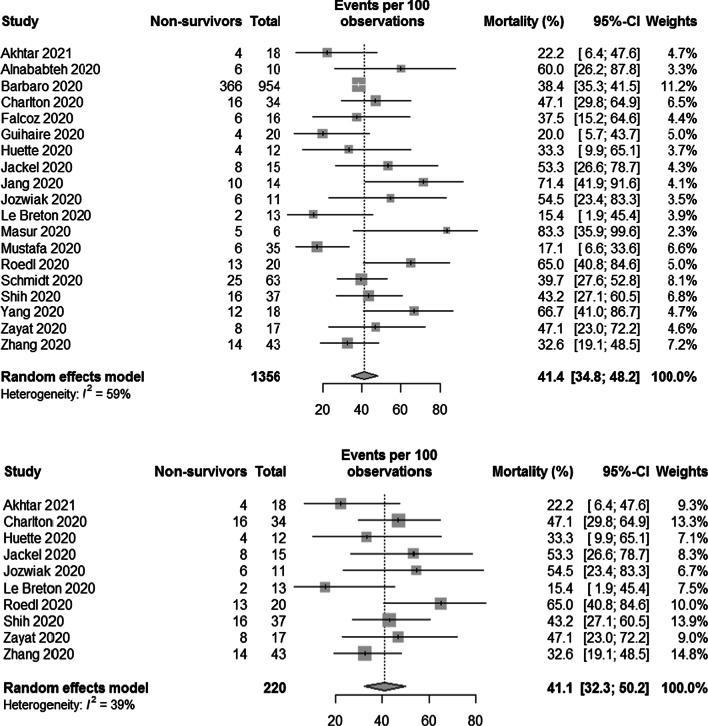
Forest plot showing the proportion of non-survivors among COVID-19 patients requiring ECMO treatment: top) omitting patients who still were on ECMO or not discharged from the hospital, bottom) omitting studies who reported on patients who still were on ECMO or not discharged from the hospital

The issue of early reporting by the underlying studies—a frequently seen phenomenon in reports on other COVID-19 cohorts—may be overcome by requesting all available follow-up data from the authors of the studies serving as input data. Calculating mortality and other outcomes based on more complete data may result in a more realistic picture of the resulting effectiveness of ECMO in patients with COVID-19 associated ARDS.

## ECMO outcomes during the COVID pandemic: Authors’ reply

**To the Editor,**

We thank Hoechter et al. for their insightful comments and for highlighting the discrepancy between Table 1 and Figure 2 regarding the number of patients included from the Japanese National Database. That database detailed 370 patients supported with extracorporeal membrane oxygenation (ECMO), of whom 120 patients died. There were 343 (93%) patients who received venovenous ECMO and 111 (32%) of them died. The overall pooled mortality remains unchanged (37%). Upon reanalysis, the pooled mortality for those who received venovenous ECMO as well as the regional mortality in Asia also remained largely unchanged at 36% (31–41%), and 43% (29–58%), respectively.

We agree with Hoechter et al. that the issue of early reporting of studies included in our meta-analysis is worthwhile. We note that the authors recalculated the primary outcome and reported a pooled mortality rate of 41% after excluding both patients who were still being treated in hospital and those who remained on ECMO. We acknowledge that reporting outcomes in patients for whom the final disposition (e.g., death or hospital discharge) is not known has the potential to either underestimate or overestimate survival. However, given the constraints the authors of the primary articles were working under and the need for urgent scientific analysis during the pandemic, some degree of incompleteness may have been unavoidable. We chose in-hospital mortality as our primary outcome, while acknowledging as a limitation the fact that some patients were still receiving ECMO or remained in hospital [1]. The true mortality may lie somewhere in between what our review demonstrated (37%) and what Hoechter et al. calculated (41%).

Finally, while either figure may be reassuring given the very high mortality reported with ECMO at the outset of the pandemic, there are reasons to be concerned that outcomes after ECMO support may have considerably worsened later on in the pandemic and a more updated analysis will be warranted [4]. The Extracorporeal Life Support Organization COVID-19 Registry reports an in-hospital mortality rate of 48% for the 6638 patients with confirmed COVID-19 who were initiated on ECMO at least 90 days earlier (accessed July 13, 2021). [5] This should be interpreted with caution because up to 27% (1792/6638) of patients may still be in hospital at 90 days. Nonetheless, outcomes from COVID-19 after ECMO support should be seen as dynamic and decision-making regarding ECMO candidacy should evolve alongside the reported outcomes.

## Data Availability

All data generated or analysed during this study are included in the published studies and their supplementary information files.
